# Exploring the effects of suboptimal affective priming: enhancement and minimization

**DOI:** 10.3389/fpsyg.2014.00499

**Published:** 2014-05-23

**Authors:** Dorota Karwowska, Dorota Kobylińska

**Affiliations:** Department of Psychology, University of WarsawWarsaw, Poland

**Keywords:** suboptimal primes, affective priming, minimization, enhancement, automatic and reflective system

There is a substantial body of knowledge regarding the influence of implicit information on human cognitive processes and behavior. Influence of this type of information has been repeatedly noted in research on evaluation processes (e.g., Zajonc, 1980; Murphy and Zajonc, 1993), stereotyping (Bargh, 1999), and behavior (e.g., Bargh et al., 1996), as well as in many other areas. Many researchers emphasize the fact that information acting outside of the conscious awareness considerably affects human functioning. Nonetheless, still little is known about factors modifying and reducing this influence. In the opinion article, our aim is to analyse various factors which could potentially increase the influence of implicit information on human functioning as well as those which may lead to modification and reduction of this influence.

## The evidence for enhancement and minimization of the suboptimal priming effect

Previous studies by Kobylińska (2001), using the affective priming paradigm indicate an existence of two types of influence exerted by affective information on making judgments: the assimilation effect and the contrast effect. Occurrence of these effects is not random and is associated with important psychological and neurobiological processes taking place during affect induction. Researchers assume that the assimilation effect is related to a strong influence of unconscious processes, unmediated by interactions associated with cognitive processing (Glaser and Banaji, 1999). In the case of the contrast effect, the direction of evaluation is opposite to the direction of suboptimal stimulation. Thus, it is assumed that its occurrence is mediated by interactions between affect and cognition. One of frequently considered factors in regulation of the influence of suboptimal stimuli on evaluations is the system of information processing, operating at the moment of making a judgment (Karwowska, 2007; Jarymowicz, 2009). Theorists emphasize that activation of one of the two systems of information processing—automatic (impulsive) or reflective—may significantly influence the process of judgment making (e.g., Epstein, 1991; Strack and Deutsch, 2004). Evaluation of the world with simultaneous activation of the automatic system is susceptible to various influences, including the influence of suboptimal stimuli. This system is evolutionarily more primitive and it is controlled by primitive brain structures. Its main role is to ensure survival of the individual and their effective adaptation to the environment. Its functioning is based on fast, automatic reactions, often without the engagement of attentional processes. As it does not consume cognitive resources, it optimizes the individual's functioning. On the other hand, the reflective system engages attention and conscious awareness in the analysis and evaluation of reality. This system is evolutionarily younger and is associated with reactions and world evaluations using knowledge and cognitive processing. From this perspective, it is more rational and less susceptible to various influences. Researchers propose that depending on which of the two systems is more readily available, evaluation can be influenced to a larger or to a lesser degree by suboptimal stimuli.

Theorists are inclined to believe, for example, that evaluation occurring under the influence of the automatic system may be determined by unavailability of cognitive resources (e.g., Epstein, 1991; Strack and Deutsch, 2004; Kolańczyk, 2007). Thus, situations in which the individual is subjected to time pressure or cognitive overload should increase the influence of affective suboptimal stimuli. Indeed such effects were observed in numerous studies (Kolańczyk, 2007). In turn, engagement of the reflective system in world evaluation may significantly modify the influence of this type of stimuli through increased attention and cognitive processing, engaged in the analysis of the object of the evaluation. Currently researchers disagree about which particular factors facilitate either the stronger influence of suboptimal stimuli or their modification or reduction. Some suggest that the main factor responsible for modification is the engagement of conscious awareness (e.g., Strack et al., 1993). However, as some data shows, awareness of the influence alone, albeit it facilitates its modification, does not lead to a reduction in susceptibility to this influence.

For example, in Karwowska's study (2005), participants were informed that suboptimal stimuli would be displayed before the neutral stimuli (Japanese ideograms), but they were not informed about the nature of these stimuli. The study used photographs of faces expressing joy or disgust, presented for 8 ms. Participants were asked to evaluate the displayed ideograms on a scale. Results have shown that susceptibility to the influence of implicit stimuli was the same in the group not informed about the suboptimal stimuli as in the informed group. However, the only difference between the groups was associated with the direction of influence of the affective stimuli. In the case of the uninformed group, participants rated neutral stimuli primed with positive affective stimuli as more positive than stimuli primed with negative affective stimuli (assimilation effect). In the informed group this influence was modified and we obtained a reversed pattern of results (contrast effect). The above results suggest that awareness of the influence of suboptimal stimuli alone does not reduce susceptibility, but leads only to modification of the influence.

Some theorists suggest that one of the factors playing an important role in increasing or reducing influence of suboptimal primes is availability of attention (De Houwer and Randell, 2002; Kolańczyk, 2007). Several experiments (e.g., Kolańczyk, 2007), in which either availability of attentional resources or the type of attentional process was manipulated before using the affective priming paradigm, have shown differences in the influence of suboptimal stimuli on evaluations. In conditions burdening attention assimilation effects were found. On the other hand, in conditions not burdening attention contrast effects were observed. Unfortunately, these studies did not compare susceptibility to implicit influence.

It is also assumed that the presence of cognitive processing alone, even of the most elementary kind, may modify or reduce the influence of suboptimal stimuli (e.g., Murphy and Zajonc, 1993). In Kobylińska's (2001) research this assumption was explored focusing on situational factors related to more complex information processing. The assumption was that activation of elementary cognitive processing might lead to modification of automatic assimilative influence of suboptimal affective stimuli. The lengthening (from 4 to 16 ms) of the exposure duration of affective primes (faces expressing joy and disgust) resulted in obtaining the contrast effect. Thus, the results did not show any differences with regard to the strength of the influence of the implicit affective stimuli. However, they did show a different direction of influence of the suboptimal stimuli on evaluations of neutral targets. Furthermore, researchers (Godlewska and Ohme, 2001) explored the issue of a more specific activation of cognitive processes, in this case associated with the type of evaluation participants were asked to perform. It was assumed that the more the evaluation engages the process of analysis of the target object, the higher the probability of occurrence of modification effects of the initial affective reaction induced by the suboptimal stimuli.

A study by Godlewska and Ohme (2001) manipulated the type of judgment participants were asked to make while evaluating neutral stimuli. It used two types of instructions: related to preferences (“I don't like it – I like it”) or associated with increased cognitive engagement (it represents “chaos/conflict – harmony/order”). The results have shown that in the case of instructions related to preferences an assimilation effect was found, while in the case of the cognitive instruction a contrast effect was observed. The results of this study indicate that the effects of the influence of implicit information differ according to the level of complexity of the judgment the individual is asked to make.

In search of factors not as much modifying, as reducing the influence of suboptimal stimuli, we focused on one of the features of the reflective system (Karwowska, 2001). This time we decided to activate reflective evaluation. It was presumed that analysing positive and negative aspects of a social phenomenon before the suboptimal stimuli manipulation will result in a lowered susceptibility of the participants to this type of influence. Therefore, in Karwowska's (2007) study, before the affective priming procedure, participants were asked to think about and to note down arguments regarding positive and negative consequences of patriotic attitudes. In the control condition participants did not perform any tasks before the cognitive priming. However, in order to examine whether modification of the influence might be due to activation of thinking processes through an additional task alone, one more experimental condition was added. In this condition participants performed a simple cognitive task consisting of comparing numbers and symbols. Results have shown a strong contrast effect in control condition as well as in the condition where participants compared symbols and numbers, while no effect of the influence of suboptimal priming was found in the condition of activation of reflective evaluation. A comparison of the degree of influence across conditions indicated significantly lower influence of suboptimal stimuli in the condition of activation of reflection than in the other two conditions. Moreover, similar results of reducing the influence of suboptimal affective primes during the activation of emotional control standards (also related to reflective system of evaluation) was found in Kobylińska's (2001) study. In this study the minimization of the prime influence on judgments was specific and occurred when negative primes were exposed in the left visual field (directed to the right hemisphere) and positive affective primes exposed in the right visual field (directed to the left hemisphere).

## Conclusions

The research findings reviewed in this opinion article allow us to derive certain conclusions regarding factors which play a role in increasing, modifying or reducing the influence of suboptimal stimuli on evaluations. Although researchers disagree about which particular factors are the most important for increasing or reducing the influence of suboptimal stimuli on evaluations, they nonetheless seem to agree that these influences are dependent on the engagement of two systems of information processing and world evaluation.

We conclude that dominance of the automatic system in the evaluation process will lead to an increase in the strength of the influence of implicit information. An evidence for this would be a direction of evaluation consistent with the direction of the influence of suboptimal stimuli—the so-called assimilation effect. On the other hand, the engagement of the reflective system will change or reduce this influence, leading to modification of evaluations and a reduction in the strength of the effect.

### Conflict of interest statement

The authors declare that the research was conducted in the absence of any commercial or financial relationships that could be construed as a potential conflict of interest.
